# Nasogastric feeding intolerance in the critically ill

**DOI:** 10.1186/cc9799

**Published:** 2011-03-11

**Authors:** S OConnor, J Rivett, A Poole, A Deane, K Lange, R Yandell, Q Nguyen, R Fraser, M Chapman

**Affiliations:** 1 Royal Adelaide Hospital, Adelaide, South Australia, Australia; 2 University of Adelaide, South Australia, Australia; 3 Repatriation Hospital, Adelaide, Australia

## Introduction

The aims of this study were to determine when patients develop feed intolerance, the prevalence of feed intolerance in subgroups, and other factors that influence feed intolerance. Nasogastric delivery of nutrition commonly fails in critically ill patients. However, studies to date have been underpowered to formally define the determinants of feed intolerance.

## Methods

A prospective observational study. Data were collected for 14 days (or until ICU discharge/or death) after commencement of gastric feeding in consecutive, ventilated patients. Gastric aspirates were performed 6 hourly. Feed intolerance was defined as ≥1 gastric aspirate(s) ≥250 ml. Data are presented as median (range). The association between feed intolerance and LOS was calculated using the Mann-Whitney U test. The ANCOVA test was used to test for a difference between groups in LOS adjusting for covariates.

## Results

In 214 patients (138 male:76 female, 56 (18 to 90) years, APACHE II 21 (5 to 46), ICU LOS 9 (1 to 94) days, hospital LOS 29 (3 to 177) days), feed intolerance occurred in 78 (37%). The first occurrence of feed intolerance was within 5 days of commencing feeding (97%). Patients with trauma (60%), traumatic brain injury (57%) and sepsis (42%) had higher incidence of intolerance than the total population. The neurological group had significantly lower incidence of intolerance (17%; *P *= 0.02). Prokinetics were administered to 29%; duration 1 (1 to 7) day. Feed intolerance was not associated with ICU or hospital mortality (ICU; intolerant 48% vs. tolerant 52% died, *P *= 0.08: hospital; intolerant 40% vs. tolerant 60% died, *P *= 0.31), but was associated with longer ICU and hospital LOS (ICU; intolerant 13 (1 to 94) days vs. tolerant 7 (1 to 51) days, *P *≤0.001: hospital; intolerant 32 (10 to 120) days vs. tolerant 26 (3 to 177) days, *P *= 0.02). There was no difference in APACHE II score between intolerant and tolerant groups (intolerant = 23 (7 to 46), tolerant = 21 (5 to 35), *P = *NS).

## Conclusions

The majority of feed intolerance occurred early in the patient's illness. While mortality was unaffected, ICU and hospital LOS were longer in feed-intolerant patients that were not explained by severity of illness on admission. Further research is needed to determine whether increasing calorie delivery improves clinical outcomes in feed-intolerant patients.

